# Predictive utility of systemic inflammatory markers in differentiating laryngeal lesions

**DOI:** 10.1007/s00405-025-09550-2

**Published:** 2025-07-08

**Authors:** Hasan Çanakcı, Kamil Gökçe Tulacı, Burak Özden, Erhan Arslan, Haşmet Yazıcı, Tuğba Tulacı, Omer Hizli

**Affiliations:** 1 Department of Otolaryngology, Faculty of Medicine, Balikesir University, Balikesir, Türkiye; 2 Etlik City Hospital, Ankara, Türkiye; 3 Private Practice, Balikesir, Türkiye

**Keywords:** Systemic inflammatory marker, Laryngeal lesion, SII, SIRI, HALP

## Abstract

**Purpose:**

Laryngeal lesions encompass both benign and malignant pathologies and present with symptoms ranging from hoarseness to throat pain. This study aimed to evaluate the predictive value of systemic inflammatory markers, including neutrophil-to-lymphocyte ratio (NLR), lymphocyte-to-monocyte ratio (LMR), platelet-to-lymphocyte ratio (PLR), systemic immune-inflammatory index (SII), systemic inflammatory response index (SIRI) and haemoglobin-albumin-lymphocyte-platelet (HALP) score, for distinguishing malignant from benign laryngeal lesions.

**Study design:**

Single-center retrospective cross-sectional analysis.

**Setting:**

Tertiary medical institution.

**Methods:**

This single-centre retrospective cross-sectional study analysed 442 patients presenting with hoarseness at a tertiary centre between 2020 and 2023. Based on their biopsy results, the patients were classified into four groups: benign lesions, low-grade dysplasia, high-grade dysplasia, and squamous cell carcinoma (SCC). Preoperative blood tests were used to calculate inflammatory markers. Statistical analyses included Kruskal–Wallis tests and post-hoc pairwise comparisons, with significance set at *p* < 0.05.

**Results:**

The SCC group demonstrated significantly higher median NLR, SII and SIRI values and lower LMR and HALP scores than the benign and dysplastic groups (*p* < 0.001). Among the indices, SIRI exhibited the highest predictive accuracy for SCC, with an AUC of 0.7, followed by NLR (0.681) and SII (0.672). HALP showed predictive value but was significant only between SCC and benign lesions. Multivariate logistic regression identified SII and LMR as predictive factors.

**Conclusion:**

Systemic inflammatory markers, particularly SIRI, SII and LMR, hold promise as adjunctive diagnostic tools for assessing malignancy risk in laryngeal lesions. Although their standalone prognostic utility remains limited, these indices may improve clinical decision-making by identifying high-risk patients for earlier biopsy appointments.

## Introduction

Laryngeal hyperplastic diseases present a spectrum of conditions ranging from benign lesions to squamous cell carcinoma (SCC). Early and accurate diagnosis is crucial for effective treatment planning and prognosis. However, traditional diagnostic methods, such as physical examination, laryngoscopy and biopsy, can be time-consuming and invasive. In recent years, systemic inflammatory markers and indices have emerged as potential noninvasive biomarkers for the diagnosis and prognosis of various cancers [1–3]. These markers include parameters such as white blood cell count (WBC), C-reactive protein (CRP), erythrocyte sedimentation rate (ESR) and platelet count.

Inflammatory markers are elevated in cancer patients, indicating an inflammatory response. For instance, high CRP levels and increased ESR have been associated with poor prognosis in various malignancies [4]. In the human body, the tissue-level inflammatory response represents the initial defence against pathological events, leading to fluctuations in the counts of various inflammatory cells. Specifically, neutrophils accumulate in tumoral tissues through mechanisms that induce genetic damage, accelerate tumorigenesis and activate angiogenesis. Conversely, lymphocytes—key players in antitumor immunity—often decrease in many different cancer types. Platelets, in tandem with neutrophils, enhance tumor angiogenesis, increase microvascular permeability and facilitate tumor cell extravasation. Monocytes, which differentiate into macrophages, form a major component of the tumor microenvironment. In light of these shifts in inflammatory cell distributions within tumors, peripheral blood counts of neutrophils, lymphocytes, monocytes and platelets are considered to indirectly mirror the inflammatory response in the humoral tissue. Consequently, these parameters can aid in differentiating malignant lesions from their benign counterparts [5].

Systemic inflammatory indices, in contrast, include combinations of these inflammatory markers, such as the neutrophil-to-lymphocyte ratio (NLR), platelet-to-lymphocyte ratio (PLR) and lymphocyte-to-monocyte ratio (LMR). The concept of NLR, first introduced by Zahorec in 2001 [6], has been shown to provide better prognostic results than the individual components in many critical conditions. Similarly, other commonly used parameters, such as PLR, LMR and the systemic immune-inflammatory index (SII), are now being used as supplementary tests for the monitoring and prognosis of specific diseases [7, 8]. These indices may provide a more comprehensive reflection of the systemic inflammatory response and immune system activity. For example, according to the available literature, an elevated NLR is associated with malignant lesions, whereas a lowered NLR is linked to benign lesions [9]. Likewise, the SII, a recently implemented parameter, offers a cost-effective means of assessing risk and prognosis in head-and-neck and other malignancies [9].

The use of systemic inflammatory markers and indices to distinguish laryngeal diseases may expedite the diagnostic process and help determine the best timing for the biopsy. In the current literature, various studies have explored the applicability of these markers and indices for early diagnosis of laryngeal cancer and for differentiating benign and malignant lesions [10, 11]. However, to the best of our knowledge, no studies have yet investigated the association between laryngeal cancer and the haemoglobin–albumin–lymphocyte–platelet (HALP) score, a widely studied prognostic parameter in previous research on inflammatory diseases and various cancer types [12].

In this study, our aim was to investigate the predictive value of systemic inflammatory markers for distinguishing malignant lesions in laryngeal diseases. We performed a comparison of various systemic inflammatory markers, including NLR, LMR, PLR, SII, systemic inflammatory response index (SIRI) and HALP score, in patients with benign lesions, low-grade dysplasia, high-grade dysplasia, and SCC.

## Materials and methods

### Subjects and design

This cross-sectional study was conducted following approval from the Institutional Ethics Committee of B******* University (IEC Number: 2023/122) and adhered to the principles of the Declaration of Helsinki. Informed consent was obtained from all participants. A total of 442 patients who presented with hoarseness at our tertiary institution between 2020 and 2023 were included.

Preoperative blood tests collected before biopsy were analysed to calculate the following inflammatory markers: NLR, LMR, PLR, SII [(neutrophil count × platelet count)/lymphocyte count], SIRI [(neutrophil count × monocyte count)/lymphocyte count], and HALP score [haemoglobin (g/L) × albumin (g/L) × lymphocytes (/L)/platelets (/L)].

The study included adult patients (≥ 18 years) diagnosed with laryngeal hyperplastic lesions via direct laryngoscopy and subsequent biopsy. Exclusion criteria were a history of systemic inflammatory diseases, chronic infections, autoimmune conditions or current use of anti-inflammatory or oncological treatments.

Based on the histopathological findings from direct laryngoscopy and biopsy, the patients were first classified into four groups: a benign lesion group (*n* = 195), a low-grade dysplasia group (*n* = 57), a high-grade dysplasia group (*n* = 36) and an SCC group (*n* = 154). The age and gender distributions of the groups were then compared. Finally, previously calculated inflammatory markers were compared across these groups to identify potential differences.

### Statistical analysis

The study results were presented as medians (min–max) and percentages (%). The normality of the data distribution was evaluated using the Kolmogorov-Smirnov test, confirming that the data did not follow a normal distribution (*p* < 0.05). The Kruskal-Wallis test was employed to compare data across the four groups, followed by post-hoc tests for pairwise comparisons. Bonferroni correction was applied to maintain a significance threshold of *p* < 0.05 for pairwise group comparisons. The predictive factors of SCC were investigated using univariate and multivariate logistic regression analyses. Variables with primary comparison values of *p* < 0.25 were included in the univariate logistic regression model, while variables with values of *p* < 0.1 in the univariate logistic regression analysis were included in the multivariate logistic regression model. Hosmer–Lemeshow goodness-of-fit statistics were used to evaluate the model fit. Cox and Snell pseudo-R2 and Nagelkerke pseudo-R2 tests were used to assess the consistency between the variables. Statistical analyses were performed using SPSS software for MacOS (SPSS Inc., Chicago, IL). Values of *p* < 0.05 were considered statistically significant.

## Results

A total of 442 patients were enrolled in this study. Table 1 presents the age and gender distributions of the study groups. Significant differences were observed among the four groups in terms of both age and gender (*p* < 0.001). While no significant age difference was found between the SCC and high-grade dysplasia groups (*p* = 1), the median age was significantly higher in the SCC group than in the benign lesion and low-grade dysplasia groups (*p* < 0.001). Similarly, the median age was significantly higher in the high-grade dysplasia group than in the benign and low-grade dysplasia groups (*p* < 0.001). Male gender predominance was observed in all groups, with the highest proportion of male patients found in the high-grade and SCC groups (94.6% and 90.3%, respectively).

**Table 1:** Distribution of patients by age and gender across the groups

	Age [median(min-max)]	Male [*n*(%)]	Female [*n*(%)]	Total [*n* (%)]
Benign	49 (17–89)	126 (64.62%)	69 (35.38%)	195 (100%)
Low grade	55 (32–80)	42 (73.68%)	15 (26.32%)	57 (100%)
High grade	64 (52–84)	34 (94.44%)	2 (5.56%)	36 (100%)
SCC	65 (42–92)	139 (90.26%)	15 (9.74%)	154 (100%)
P* value	**< 0.001**^**#**^	<0.001*		-

*p < 0.05 was considered significant, ^#^ *p* value of Kruskal Wallis test, * *p* value of Chi- square test

Kruskal–Wallis analysis revealed statistically significant differences among the four groups for the median NLR, LMR, SII, SIRI and HALP values (*p* < 0.001). However, no statistically significant difference was evident in the median PLR values among the groups (*p* = 0.093) (Table 2).

**Table 2:** Comparison of NLR, PLR, LMR, SII, SIRI and HALP across four groups revealing the significant differences of NLR, LMR, SII, SIRI and HALP

	Benign lesion (*n*:195) [median(min-max)]	Low grade (*n*: 57) [median(min-max)]	High grade (*n*: 36) [median(min-max)]	SCC (*n*: 154) [median(min-max)]	P* value
NLR	2 (0.89–12.30)	2.13 (0.22–12.78)	1.97 (0.95-20)	2.53 (0.92–13.5)	**< 0.001**
LMR	3.80 (1-10.33)	4.17 (1.43-10)	3.29 (0.45–6.40)	3.06 (0.67-7)	**< 0.001**
PLR	117.65 (48.39-555.71)	107.89 (49.50-627.5)	116.83 (38.70–552)	127.17 (46.58-631.43)	0.093
SII	505.80 (169.35-2619.90)	569.25 (50.97-1750.56)	492.77 (131.57–8004)	659.93 (177-5872.30)	**< 0.001**
SIRI	1.20 (0.28–8.06)	1.17 (0.19–2.87)	1.33 (0.39–31.90)	1.62 (0.42–16.38)	**< 0.001**
HALP score	5.48 (0.73-106.21)	4.86 (0.87–12.25)	5.22 (0.74–12.53)	4.06 (0.47–12.38)	**< 0.001**

*p < 0.05 was considered significant; *NLR* Neutrophil- Lymphocyte ratio; *LMR* Lymphocyte-monocyte ratio; *SII* Systemic inflammation index; *SIRI* Systemic inflammatory response index; *HALP score* [hemoglobin (g/L) × albumin (g/L) × lymphocytes (/L)]/platelets (/L)

Table 3 shows the pairwise comparisons for the groups. The median NLR value was significantly higher for the SCC group than for the benign lesion, low-grade dysplasia, and high-grade dysplasia groups (*p* < 0.001, *p* = 0.023 and *p* = 0.023, respectively). The median LMR value was significantly lower for the SCC group than for the benign lesion and low-grade dysplasia groups (*p* < 0.001). The median SII value was significantly higher for the SCC group than for the benign lesion, low-grade dysplasia, and high-grade dysplasia groups (*p* = 0.001, *p* = 0.039, and *p* = 0.021, respectively). The median SIRI value was significantly higher for the SCC group than for the benign lesion and low-grade dysplasia groups (*p* = 0.001 and *p* < 0.001, respectively). The median HALP value was significantly lower for the SCC group than for the benign lesion group (*p* < 0.001).

**Table 3:** Statistical significance (p*-values) of pairwise comparisons between groups for NLR, LMR, SII, SIRI and HALP

	NLR	LMR	SII	SIRI	HALP score
Benign-low grade	1	0.967	1	1	1
Benign-high grade	1	0.333	1	0.929	1
Benign-SCC	**< 0.001**	**< 0.001**	**0.001**	**0.001**	**< 0.001**
Low grade-high grade	1	0.052	1	1	1
Low grade-SCC	**0.023**	**< 0.001**	**0.039**	**< 0.001**	0.864
High grade-SCC	**0.023**	1	**0.021**	**0.229**	0.883

*Bonferroni correction was applied. *p* < 0.05 was considered significant; *NLR* Neutrophil- lymphocyte ratio; *LMR* Lymphocyte- monocyte ratio; *SII* Systemic inflammation index; *SIRI* Systemic inflammatory response index; *HALP score* [hemoglobin (g/L) × albumin (g/L) × lymphocytes (/L)]/platelets (/L); *SCC* Squamous cell carcinoma

Table 4 presents the univariate and multivariate logistic regression analysis results. In the univariate logistic regression analysis, the NLR, LMR, PLR, SII, SIRI and HALP scores were predictive factors for SCC (*p* < 0.001, *p* = 0.01, *p* = 0.003 and *p* = 0.002, respectively). However, only low LMR and elevated SII were predictive factors for SCC in the multivariate logistic regression model (*p* < 0.001 and *p* = 0.004, respectively).

**Table 4:** Univariate and multivariate logistic regression analysis identifying independent predictors of squamous cell carcinoma (SCC) in laryngeal lesions. LMR and SII were finally indicated as independent predictors of SCC

	Univariate		Multivariate	
	OR (95% CI)	P* value	OR (95% CI)	P* value
NLR	1.227 (1.093–1.378)	**< 0.001**	0.828 (0.604–1.136)	0.242
LMR	0.695 (0.596–0.809)	**< 0.001**	0.805 (0.709–0.914)	**< 0.001**
PLR	1.003 (1.001–1.006)	**0.01**	0.996 (0.991–1.001)	0.115
SII	1.001 (1-1.001)	**< 0.001**	1.002 (1.001–1.004)	**0.004**
SIRI	1.256 (1.083–1.457)	**0.003**	0.827 (0.617–1.109)	0.205
HALP score	0.851 (0.768–0.942)	**0.002**	0.992 (0.94–1.047)	0.761
			Cox & Snell pseudo-R^2^ = 0.178 Nagelkerke pseudo-R^2^ = 0.238 Hosmer- Lemeshow *P =* 0.072	

*p < 0.05 was considered significant; *NLR* Neutrophil lymphocyte ratio; *LMR* Lymphocyte monocyte ratio; *PLR* Platelet lymphocyte ratio; *SII* Systemic immune-inflammation index; *SIRI* Systemic inflammation response index; *HALP* Hemoglobin (g/L) × albumin (g/L) × lymphocytes (/L)]/platelets (/L)

**Fig. 1:**
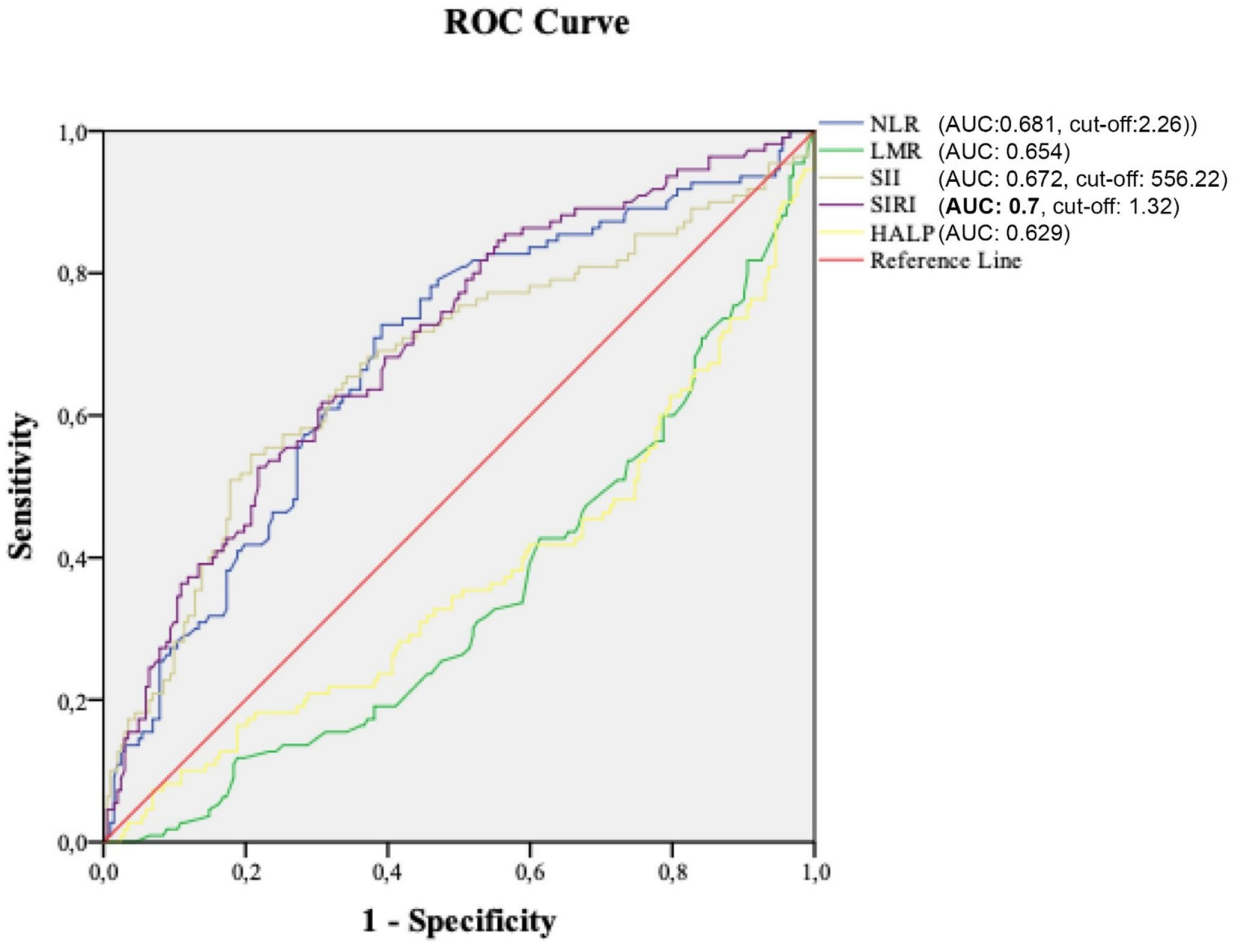
ROC curve demonstrating the predictive value of NLR, LMR, SII, SIRI, and HALP for SCC in laryngeal lesions. SIRI had the highest AUC value (bold). The top three cut-off values were identified and presented

Figure 1 illustrates the ROC curve demonstrating the predictive values of NLR, LMR, SII, SIRI and HALP for SCC in laryngeal lesions. The areas under the curve (AUC) were as follows: SIRI = 0.7 (95% CI, 0.64–0.761), NLR = 0.681 (95% CI, 0.619–0.744), SII = 0.672 (95% CI, 0.606–0.738), LMR = 0.654 (95% CI, 0.591–0.717), and HALP = 0.629 (95% CI, 0.562–0.696). The cut-off values indicating SCC in patients with laryngeal lesions were 1.32 for SIRI (sensitivity: 68%, specificity: 60%), 2.26 for NLR (sensitivity: 67%, specificity: 63%) and 556.22 for SII (sensitivity: 69%, specificity: 61%).

## Discussion

The accurate prediction of malignancy potential in laryngeal lesions before biopsy remains a critical yet challenging aspect of clinical practice. While a definitive diagnosis relies on histopathological evaluation, the ability to identify markers that can aid in estimating malignancy risk before biopsy would offer significant advantages. The availability of predictive tools could help prioritise high-risk patients for biopsy and streamline the decision-making process for both diagnostic and therapeutic interventions. In this context, our study investigated the potential role that various inflammatory markers might play in assessing the malignancy potential of laryngeal lesions, with the overall aim of optimising clinical management strategies.

Here, we classified laryngeal lesions into four categories based on biopsy results: benign lesions, low-grade dysplasia, high-grade dysplasia, and SCC. However, our demographic evaluation revealed a predominance of male gender and higher age in both the SCC and high-grade dysplasia groups, raising the possibility that this inhomogeneity may have limited the methodological robustness of the study. However, the higher prevalence of older age and male gender in the high-grade dysplasia and SCC groups is likely attributable to higher smoking rates, which is in agreement with previous literature [13, 14].

Consistent with other studies [11], our results showed a significantly higher median NLR in the SCC group than in the benign, low-grade and high-grade dysplasia groups. Recent studies have demonstrated a prognostic value for NLR in several tumor types, including oesophageal SCC and cervical, colorectal and lung cancers [15–18]. Lal et al. also reported an association between higher NLR and poor prognosis in head and neck cancers [19]. Therefore, our findings support the idea that NLR may also have prognostic value for laryngeal lesions; however, it should not be considered a standalone predictor.

We also observed a significantly lower LMR in our SCC group. This finding agrees with earlier work by Han et al., who demonstrated an association between decreased LMR and poor prognosis in patients with laryngeal cancer [10]. This association indicates that malignancy-associated inflammation significantly influences LMR, thereby supporting the potential role of LMR as a prognostic biomarker in cancer-related inflammatory processes. However, the lack of a significant difference in PLR among our study groups indicates that this biomarker may have limited value in distinguishing laryngeal lesions.

The parameters SII and SIRI have both been frequently highlighted in recent literature [20]. In the present study, both were significantly higher in the SCC group than in the benign, low-grade, and high-grade dysplasia groups. Therefore, these indices might provide a more comprehensive reflection of the inflammatory background of laryngeal lesions because they incorporate additional cell counts, such as monocytes and platelets.

The HALP score also demonstrated predictive value for SCC in laryngeal lesions, although its significance was limited when compared solely against benign lesions. The HALP score, which encompasses crucial immune and nutritional factors, such as haemoglobin, albumin, lymphocyte count and platelet count, has demonstrated prognostic value across various cancer types [12]. For instance, research on cervical cancer has shown an independent association between lower HALP scores and poor overall and progression-free survival in patients with locally advanced disease [12]. Studies on gastric cancer have also reported similar observations [12, 21]. Furthermore, in oesophageal cancer, patients with higher HALP scores exhibited improved responses to chemotherapy and prolonged progression-free survival [12]. However, studies specifically addressing the association between HALP and laryngeal lesions are noticeably lacking in the existing literature. Thus, our findings offer a novel perspective on the potential relevance of the HALP score in laryngeal pathology, while also emphasising its current exploratory status. Collectively, our findings underscore the potential of the HALP score as a readily available and comprehensive biomarker for predicting cancer outcomes. In our study, the difference in HALP scores was statistically significant only between the SCC and benign lesion groups, and its predictive value for high-grade dysplasia appeared limited. Moreover, the ROC curve analysis in the present study revealed that SIRI had the highest AUC value (0.7), followed by NLR (0.681) and SII (0.672). Nevertheless, although SIRI demonstrated the highest discriminative capacity in ROC analysis, it did not retain statistical significance in the multivariate logistic regression model that included other systemic inflammatory indices. This discrepancy likely arises from shared variance among inflammatory markers, with multicollinearity potentially obscuring the individual effects of even strong univariate predictors, such as SIRI.

In the adjusted model, low LMR and elevated SII levels emerged as independent predictors of SCC. Clinically, these findings suggest that a combined assessment of multiple inflammatory markers—especially elevated SII and SIRI along with low LMR—may help identify patients at a higher risk of SCC. Integrating these markers into pre-diagnostic workflows could guide more efficient triage strategies, including prioritisation of biopsy scheduling for those with the highest predicted SCC risk, thereby enabling earlier diagnosis and potentially improving oncologic outcomes. This would enable clinicians to consider offering earlier biopsy appointments to patients exhibiting elevated (or decreased) levels of multiple parameters—especially those showing levels above our specified cut-off values—as lesions in these individuals are more likely to harbour malignant potential. In addition, close follow-up without biopsy could be considered as an option in limited hyperplastic lesions with a lower risk profile that also remain below the specified cutoff values.

One crucial point to emphasise is that histopathological assessment remains the most definitive way to diagnose, stage and determine appropriate treatment strategies for laryngeal lesions. While systemic inflammatory indices, such as NLR, LMR, SII, SIRI and HALP, can provide valuable insights into the inflammatory milieu associated with laryngeal lesions, they should not be viewed as standalone diagnostic tools. Rather, incorporating these markers into routine clinical workflows can enhance diagnostic accuracy and efficiency. In cases where the interval between the initial consultation and laryngoscopic biopsy is extended due to high patient volume, we propose that considering patient risk factors and endoscopic findings, as well as systemic inflammatory indices, is crucial for determining biopsy urgency and prioritisation. For instance, patients with elevated marker levels—particularly those exceeding established thresholds—may warrant earlier biopsy or closer monitoring. Conversely, patients with lower levels, especially those identified as low risk based on clinical and endoscopic evaluations, may be considered for deferred biopsy or a period of observation. This inclusion of systemic inflammatory indices in the treatment approach will promote more personalised and judicious decision-making, while ensuring timely diagnosis for higher-risk individuals. Ultimately, systemic inflammatory indices should be integrated into standard diagnostic workflows to complement comprehensive clinical evaluation—including patient history, risk factors such as smoking, and laryngoscopic findings—and to guide optimal management strategies.

While the results from this study demonstrated the prognostic significance of numerous blood parameters and therefore add significantly to the currently limited literature, certain limitations must be acknowledged. The male predominance and higher mean age in the SCC and high-grade dysplasia groups may limit the generalisability of our findings, as these factors could be potential confounders. For instance, the lack of detailed information on smoking status—which may be associated with the higher prevalence of older age and male gender—represents an additional source of confounders, given that smoking is both a well-established risk factor for laryngeal lesions and a potential modifier of systemic inflammation. The negative effects of smoking are largely mediated through changes in inflammatory pathways, and smoking has well-established adverse effects on the hematopoietic system and lipid metabolism [22]. Numerous studies have demonstrated that smoking leads to increases in haematocrit values and in peripheral blood levels of haemoglobin, leucocytes, eosinophils and platelets, thereby elevating systemic inflammatory markers [22, 23]. Furthermore, nicotine induces the release of catecholamines and corticosteroids from the adrenal glands, resulting in an increase in leucocyte counts [22]. These physiological changes could have influenced the systemic inflammatory indices assessed in our study, particularly in the SCC and high-grade dysplasia groups, where smoking prevalence was generally higher. Gender-related differences in immune response and cancer biology may also have influenced the inflammatory marker profiles. The retrospective design of our study also inherently introduces potential biases, and the relatively lower sensitivity and specificity rates of our cut-off values might be considered another limitation of the study. In this context, we anticipate that future studies with larger sample sizes and more balanced distributions of key risk factors, such as age, gender and smoking status, will further enhance the robustness of evidence supporting the diagnostic utility of systemic inflammatory indices in differentiating laryngeal lesions. These types of studies could provide stronger statistical power and minimise confounding variables, thereby refining the predictive value of these markers in clinical practice.

## Conclusion

This study emphasises a potential role of systemic inflammatory markers and indices, such as NLR, LMR, SII, SIRI and HALP scores, for distinguishing laryngeal hyperplastic lesions and assessing malignancy risk. Among these, SIRI showed prominence by exhibiting the highest predictive accuracy for SCC when considered in conjunction with the SII and LMR values, thereby highlighting its strong potential as a prognostic indicator. These findings underscore the importance of incorporating a multi-marker approach for evaluating malignancy potential, as no single parameter can independently provide definitive prognostic information. These markers, particularly SIRI, SII and LMR, could assist clinicians in optimising biopsy timing and improving diagnostic stratification, though histopathological confirmation remains essential for definitive diagnosis and treatment planning.

## Abbreviations

NLR: Neutrophil-to-lymphocyte ratio
LMR: Lymphocyte-to-monocyte ratio
PLR: Platelet-to-lymphocyte ratio
SII: Systemic immune-inflammatory index
SIRI: Systemic inflammatory response index
HALP: Hemoglobin-albumin-lymphocyte-platelet score
SCC: Squamous cell carcinoma

## References

[1] Huang H, Liu Q, Zhu L, Zhang Y, Lu X, Wu Y, Liu L (2019) Prognostic value of preoperative systemic immune-inflammation index in patients with cervical cancer. Sci Rep 9(1):3284. 10.1038/s41598-019-39150-0 30824727 10.1038/s41598-019-39150-0 PMC6397230

[2] Wang YT, Kuo LT, Weng HH, Hsu CM, Tsai MS, Chang GH, Lee YC, Huang EI, Tsai YT (2022) Systemic immun e-inflammation index as a predictor for head and neck cancer prognosis: a meta-analysis. Front Oncol:12899518. 10.3389/fonc.2022.899518 10.3389/fonc.2022.899518 PMC9263088 35814369

[3] Zhang W, Wang R, Ma W, Wu Y, Maskey N, Guo Y, Liu J, Mao S, Zhang J, Yao X, Liu Y (2019) Systemic immune-inflammation index predicts prognosis of bladder cancer patients after radical cystectomy. Ann Transl Med 7(18):431. 10.21037/atm.2019.09.02 31700867 10.21037/atm.2019.09.02 PMC6803204

[4] Tiainen S, Nurmela V, Selander T, Turunen P, Pasonen-Seppänen S, Kettunen T, Kuittinen O, Auvinen P, Rönkä A (2023) A practical prognostic peripheral blood-based risk model for the evaluation of the likelihood of a response and survival of metastatic cancer patients treated with immune checkpoint inhibitors. BMC Cancer 23(1):1186. 10.1186/s12885-023-11699-0 38049762 10.1186/s12885-023-11699-0 PMC10694914

[5] Fang Y, Yang Y, Chen M, He P, Cheng L, Chen J, Wu H (2019) Elevated peripheral inflammatory markers are related with the recurrence and canceration of vocal fold leukoplakia. Eur Arch Otorhinolaryngol 276(10):2857–2864. 10.1007/s00405-019-05576-5 31367834 10.1007/s00405-019-05576-5

[6] Firment J, Hulin I (2024) Zahorec index or neutrophil-to-lymphocyte ratio, valid biomarker of inflammation and immune response to infection, cancer and surgery. Bratisl Lek Listy 125(2):75–83. 10.4149/bll_2024_012 38219059 10.4149/BLL_2024_012

[7] Hu B, Yang XR, Xu Y, Sun YF, Sun C, Guo W, Zhang X, Wang WM, Qiu SJ, Zhou J, Fan J (2014) Systemic immune-inflammation index predicts prognosis of patients after curative resection for hepatocellular carcinoma. Clin Cancer Res 20(23):6212–6222. 10.1158/1078-0432.Ccr-14-0442 25271081 10.1158/1078-0432.CCR-14-0442

[8] Lolli C, Basso U, Derosa L, Scarpi E, Sava T, Santoni M, Crabb SJ, Massari F, Aieta M, Conteduca V, Maruzzo M, La Russa F, Wheater M, Berardi R, Galli L, De Giorgi U (2016) Systemic immune-inflammation index predicts the clinical outcome in patients with metastatic renal cell cancer treated with Sunitinib. Oncotarget 7(34):54564–54571. 10.18632/oncotarget.10515 27409344 10.18632/oncotarget.10515 PMC5342364

[9] Sonpavde G, Pond GR, Armstrong AJ, Clarke SJ, Vardy JL, Templeton AJ, Wang SL, Paolini J, Chen I, Chow-Maneval E, Lechuga M, Smith MR, Michaelson MD (2014) Prognostic impact of the neutrophil-to-lymphocyte ratio in men with metastatic castration-resistant prostate cancer. Clin Genitourin Cancer 12(5):317–324. 10.1016/j.clgc.2014.03.005 24806399 10.1016/j.clgc.2014.03.005

[10] Han Y, Ren Z, Liu Y, Liu Y (2024) Diagnostic and prognostic value of fibrinogen, fibrinogen degradation products, and lymphocyte/monocyte ratio in patients with laryngeal squamous cell carcinoma. Ear Nose Throat J 103(5):Np278–np288. 10.1177/01455613211048970 34672822 10.1177/01455613211048970

[11] Li Z, Qu Y, Yang Y, An W, Li S, Wang B, He Y, Li J, Shao Q, Qin L (2021) Prognostic value of the neutrophil-to-lymphocyte ratio, platelet-to-lymphocyte ratio and systemic immune-inflammation index in patients with laryngeal squamous cell carcinoma. Clin Otolaryngol 46(2):395–405. 10.1111/coa.13689 33321001 10.1111/coa.13689

[12] Farag CM, Antar R, Akosman S, Ng M, Whalen MJ (2023) What is hemoglobin, albumin, lymphocyte, platelet (halp) score? A comprehensive literature review of halp’s prognostic ability in different cancer types. Oncotarget:14153–14172. 10.18632/oncotarget.28367 10.18632/oncotarget.28367 PMC9970084 36848404

[13] Park J-O, Nam I-C, Kim C-S, Park S-J, Lee D-H, Kim H-B, Han K-D, Joo Y-H (2022) Sex differences in the prevalence of head and neck cancers: A 10-year follow-up study of 10 million healthy people. Cancers 14(10):2521 35626129 10.3390/cancers14102521 PMC9139445

[14] Zhang Q-W, Wang J-Y, Qiao X-F, Li T-L, Li X (2021) Variations in disease burden of laryngeal cancer attributable to alcohol use and smoking in 204 countries or territories, 1990–2019. BMC Cancer 21(1):1082. 10.1186/s12885-021-08814-4 34620148 10.1186/s12885-021-08814-4 PMC8496083

[15] Bowen RC, Little NAB, Harmer JR, Ma J, Mirabelli LG, Roller KD, Breivik AM, Signor E, Miller AB, Khong HT (2017) Neutrophil-to-lymphocyte ratio as prognostic indicator in Gastrointestinal cancers: A systematic review and meta-analysis. Oncotarget 8(19):32171–32189. 10.18632/oncotarget.16291 28418870 10.18632/oncotarget.16291 PMC5458276

[16] Peng B, Wang YH, Liu YM, Ma LX (2015) Prognostic significance of the neutrophil to lymphocyte ratio in patients with non-small cell lung cancer: A systemic review and meta-analysis. Int J Clin Exp Med 8(3):3098–3106 26064198 PMC4443032

[17] Sürücü E, Demir Y, Şengöz T (2015) The correlation between the metabolic tumor volume and hematological parameters in patients with esophageal cancer. Ann Nucl Med 29(10):906–910. 10.1007/s12149-015-1020-4 26296613 10.1007/s12149-015-1020-4

[18] Wu J, Chen M, Liang C, Su W (2017) Prognostic value of the pretreatment neutrophil-to-lymphocyte ratio in cervical cancer: A meta-analysis and systematic review. Oncotarget 8(8):13400–13412. 10.18632/oncotarget.14541 28077792 10.18632/oncotarget.14541 PMC5355107

[19] Lal B, Thakur D, Gupta P, Dadwal M, Singh M (2024) The significance of systemic inflammatory markers in prognosis of head and neck squamous cell cancers. Indian J Otolaryngol Head Neck Surg 76(1):41–47. 10.1007/s12070-023-04070-z 38440621 10.1007/s12070-023-04070-z PMC10908702

[20] Islam MM, Satici MO, Eroglu SE (2024) Unraveling the clinical significance and prognostic value of the neutrophil-to-lymphocyte ratio, platelet-to-lymphocyte ratio, systemic immune-inflammation index, systemic inflammation response index, and delta neutrophil index: an extensive literature review. Turk J Emerg Med 24(1):8–19. 10.4103/tjem.tjem_198_23 38343523 10.4103/tjem.tjem_198_23 PMC10852137

[21] Akgül GG (2023) The relationship between the halp score and gastric cancer prognosis: halp score in gastric cancer. J Eur Intern Med Professionals 1(4):156–161

[22] Inci H, Besler MS, Inci F, Adahan D (2023) The effects of smoking cessation on the ratios of neutrophil/lymphocyte, platelet/lymphocyte, mean platelet volume/lymphocyte and monocyte/high-density lipoprotein cholesterol. Natl Med J India 36(4):224–228. 10.25259/nmji_988_20 38692637 10.25259/NMJI_988_20

[23] Gumus F, Solak I, Eryilmaz MA (2018) The effects of smoking on neutrophil/lymphocyte, platelet/ /lymphocyte ratios. Bratisl Lek Listy 119(2):116–119. 10.4149/bll_2018_023 29455548 10.4149/BLL_2018_023

